# Emerging Data from a Newborn Hearing Screening Program in Sharjah, United Arab Emirates

**DOI:** 10.1155/2021/2616890

**Published:** 2021-06-27

**Authors:** Muhammed Ayas, Hakam Yaseen

**Affiliations:** ^1^Audiology Unit, University Hospital Sharjah, UAE; ^2^Clinical Sciences College of Medicine, University of Sharjah, UAE; ^3^Neonatology & Pediatrics, University Hospital Sharjah, UAE

## Abstract

**Objectives:**

Newborn hearing screening (NHS) plays a critical role in early identification of hearing loss and subsequent early habilitation. Active parental involvement influences the success of NHS, particularly the initial NHS and follow-up. The current study reports the results of an NHS program in a cohort of babies born in a tertiary care hospital in Sharjah, United Arab Emirates (UAE). Further, it explores a two-stage NHS model to reduce false responses, thereby alleviating parental anxiety.

**Methods:**

Retrospective observational study was conducted for a period of five years from January 2017 to December 2020. NHS was done as a two-stage model. All the healthy newborn babies were screened using Automated Auditory Brainstem Response (AABR) by trained audiology professionals. Babies who failed the first NHS were followed up after two weeks. Further, babies that failed the follow-up NHS were sent for diagnostic hearing evaluation and intervention as necessary.

**Results:**

A total of 1821 newborn babies were screened during the study period. Eighty-one percent of babies passed the initial NHS. Four hundred and twenty-three (23.22%) babies were referred on the first NHS and were followed up after 2 weeks. Among these babies, 7.03% (24) failed second NHS. Nine (37.50%) of the 24 babies were confirmed to have hearing loss in both ears. The incidence of hearing loss in our cohort was 4.94 per 1000. Confirmed hearing loss was statistically higher in boys than girls (*p* < 0.05)

**Conclusion:**

Current study was an attempt to report the emerging NHS data as part of the implementation of an NHS program. The study findings emphasize the need for a two-stage model of NHS to rule out false responses.

## 1. Introduction

Newborn hearing screening (NHS) has significantly lowered the age for early detection of hearing loss in children [1]. Hearing loss at birth has been reported in approximately 1 to 3 neonates per 1000 births [2]. Joint Committee on Infant Hearing (JCIH) announced its position statement in 1994 supporting early detection and identification of hearing loss in infants. It also proposed the improvement in the methods used for assessment and intervention in those identified with hearing loss [3–5]. In 2002, 2007, and recently in 2019, the position statement has been updated. However, the core aim of JCIH remains the same. It emphasizes the processes established for the successful implementation of NHS in various settings.

Over the past 25 years, several countries have successfully implemented NHS programs. However, all the NHS programs faced significant bottlenecks with processes at initiation [6–8]. Primarily, there is a shortage of trained professionals to lead the program. These staffing issues have adversely affected the follow-up surveillance program for infants in the high-risk category. These infants are vulnerable to developing hearing loss, irrespective of their initial NHS test [9, 10]. The 2019 JCIH position statement highlights the need for audiology-driven NHS programs. Trained audiologists are more qualified to deal with parents, other health professionals, and community workers [11].

NHS is generally an inexpensive procedure that does not incur major costs. The test is easy to administer, if the clinical professionals performing the test have adequate training and competency [12]. It is also essential for the professionals to maintain the screening equipment with up to date calibration [13]. Limited parental involvement and lack of awareness regarding hearing loss in children have been hindering the success of NHS programs. Many countries have initiated various methods in creating enhanced level of awareness among the mothers, extended family members, and caregivers [14–16]. Findings in literature suggest that 25% of hearing loss is reported during the postnatal phase [17]. This emphasizes the importance of NHS programs in detecting childhood hearing loss.

In the United Arab Emirates (UAE), there are limited data reported on NHS programs. One study compares healthy newborns and neonatal intensive care unit- (NICU-) admitted infants. The study reports that healthy newborns have higher incidence of hearing loss than in NICU-admitted infants [18]. Therefore, there is a dire need for developing and implementation of NHS in all hospital-based settings.

The current study is aimed at reporting the results of a NHS program in a cohort of babies born in a tertiary care hospital setting in Sharjah, UAE, as a part of the nationwide implementation. It also explores a two-stage NHS model in reducing false referrals' responses thereby alleviating the parental anxiety.

## 2. Method

### 2.1. Participants

Retrospective observational study design was used for the current study. The study was carried out in the NHS unit at the University Hospital Sharjah, UAE. The study period was from January 2017 to December 2020. The study included all healthy newborn babies who underwent NHS after written signed informed consents were taken from the parents. All the procedures performed in the study were in accordance with the ethical standards of the institutional research committee and in accordance with the 1964 Helsinki declaration. The study was approved by the hospital research and ethics committee (UHS-HERC-041-03012021).

### 2.2. NHS: Initial Screening

NHS was done on all healthy newborn babies on the second day after birth or just before discharge from the hospital. The test was carried out in the NHS unit by using duly calibrated Natus Algo-3i (Natus Medical Inc., Pleasanton, CA, USA) Automated Auditory Brainstem Response (AABR) equipment. The results read “Pass,” if the newborn baby clears NHS. If the responses were not adequate, the results read “Fail.” The test results were then relayed by the audiologist to the parents who were present in the unit during the procedure. Further, the results were documented in the hospital electronic medical record. After NHS, the babies were transferred back to the room with the mothers.

### 2.3. NHS: Follow-Up Screening

If the responses for AABR were “Fail” for one or both ears, the parents were informed and given a repeat appointment after 2 weeks. The same procedure was performed during the follow-up visit. If the results obtained were “Pass” in both ears, parents were informed and were advised to monitor the auditory milestones at home. If the results were the same as the first NHS or if any variations were noted in laterality, the parents were counseled by the audiologist regarding the test outcome. They were also educated on the need for diagnostic hearing assessment.

After the necessary approvals were taken, the babies were given appointments for diagnostic ABR (Auditory Brainstem Response) and OAE (Oto-Acoustic Emissions) with an audiologist in the audiology clinic at the hospital. Diagnostic procedures were performed to confirm the hearing status and to conclude the final diagnosis of the severity and type of hearing impairment. Further, the babies were advised to be enrolled for rehabilitation.

### 2.4. NHS Procedure

NHS was done by using a calibrated AABR equipment. Before the procedure, the newborn baby was fed to aid in ensuring that the baby was well settled for the test. In order to prepare the newborn baby for AABR, the baby's skin surface was gently cleaned with the skin prep gel to increase conduction and to reduce the resistance. This further improves the electrode impedance ensuring better screening results. The disposable surface electrodes were placed on the forehead, nape, and upper shoulder to accurately record the responses. An intensity level of 35 dBnHL was presented to both the ears simultaneously. Once the procedure attained a particular sweep level, it matched the inbuilt analyzed data. This was a preprogrammed normative within the system that delivered the results.

### 2.5. Statistical Analysis

The data was entered and analyzed using Statistical Package for the Social Sciences (SPSS, version 20, Chicago, IL, USA). All the obtained data were analyzed descriptively and expressed in percentages. To assess the data trait and to explore the effect of dichotomous homogeneity of the “Pass” and “Fail” results in initial and follow-up NHS, a McNemar's chi-square test was done. A *p* value of less than 0.05 was considered statistically significant.

## 3. Results

A total of 1821 healthy newborn babies were screened for their hearing status during the study period. Among these, 1165 (64%) were females and 656 were (36%) males. The gestational age of the cohort was 37.2 ± 2.2 weeks, and birth weight is 2.86 ± 0.76.

During the initial NHS procedure, the laterality of the AABR responses was 1592 (87.40%) “Pass” for the right ear and 229 (12.60%) “Fail.” For the left ear, 1627 (89.30%) were “Pass” and 194 were (10.70%) “Fail.” Four hundred and twenty-three (23.22%) babies who failed the initial NHS were followed up after 2 weeks. Twenty-four (05.70%) out of 423 babies were found to be “Fail” in both ears (Table 1).

All 24 babies were referred for diagnostic audiological evaluation to assess their hearing status. Nine (37.50%) of the 24 babies had confirmed hearing loss in both ears. The incidence of hearing loss in our cohort was 4.94 per 1000. Regarding gender distribution, confirmed hearing loss was higher in boys than girls (*p* < 0.05). Fifteen (62.507%) babies showed mixed responses in diagnostic ABR and OAE. Of the 15 babies, 12 showed either delayed peak latencies or changes in threshold levels and 03 babies' results revealed auditory neuropathy spectrum disorders (ANSD) (Table 2).

McNemar's chi-square test results comparing the right ear and left ear results are given in Tables 3 and 4. The data set responses showed that there were statistically significant results, when the initial NHS data was compared with follow-up NHS results after two weeks (*p* < 0.001). Those babies who were followed up after 2 weeks for repeat NHS had significantly less number of “Fail” results.

## 4. Discussion

The current study is aimed at reporting the results of an NHS program in a cohort of babies born in a tertiary care university hospital in Sharjah, UAE. Based upon our literature review, these findings from the two-stage NHS are the first to be reported in UAE. The study attempted a two-stage NHS model in order to reduce false “Fail” responses during NHS.

The relatively high percentage (23.22%) of false “Fail” rates questions the potential negative influence of NHS on the parents. This usually leads to increased anxiety levels of the parents and unnecessary referrals for audiological evaluation. It is noted that majority of the parents of children with hearing loss are psychologically distressed. In the current study, it was observed that parents were anxious during their baby's NHS procedure. Previous studies in literature report numerous patterns of emotional response from the parents to “Fail” results of their child, including anger, frustration, helplessness, and denial [19, 20].

In the current study, care was taken to manage the emotional strains on the parents while informing their child's “Fail” results. It is reported in literature that mothers usually go through psychological stress and postpartum depression following maternity and labor [21, 22]. In the current study, the audiologists focused on a clinical and professional approach in relaying the information to the parents. We believe that this may have reduced the anxiety levels among parents while accepting the “Fail” results. The parents were also informed regarding the significance of follow-up NHS.

Further, in our study, the follow-up NHS was exclusively performed by audiologists. The parents were counseled in detail to help reduce their anxiety, before initiating the procedure. Details regarding the test process, possible false “Fail” responses and auditory milestones were provided to the parents in advance [23, 24]. Majority of the parents were anxious about the outcomes and feared that their child may have permanent hearing loss. “Fail” test results during the follow-up indicate that their child may have hearing loss.

The incidence of hearing loss in the current cohort was 4.94 per 1000. This concurred with the reported data in literature [25, 26] that many countries who introduced NHS could detect 1-3 permanent childhood hearing loss in 1000 live births. In the current study, 09 babies showed severe to profound hearing loss. They were fitted with appropriate amplification. At this stage, parents were counseled by the audiologists and emotionally prepared to take on the habilitation process, including cochlear implantation [27]. Though parental readiness is overlooked during diagnostic NHS, it is believed that parental preparation is critical to the success of hearing habilitation through hearing aids or cochlear implants [28, 29].

Interestingly, our study findings demonstrated that 15 (62.50%) babies failed in the follow-up NHS and showed mixed responses in diagnostic hearing evaluation. They were categorized to have auditory maturation delay or were consistent with ANSD. From our study, the incidence of ANSD was 1.65 in every 1000 infants. However, it was significantly higher than existing reported data [30–32].

### 4.1. Implications

One of the key aspects that were successful in the current NHS program was that it followed a two-stage model of NHS. The study findings emphasize the need for a two-stage model NHS. Although a high percentage of results was reported “Fail” during the initial NHS, parental counseling by audiologists helped to increase the uptake of the follow-up NHS. This aided in minimizing the false responses and in achieving early hearing impairment and interventions for the babies.

### 4.2. Limitations

Though the current study reported data from a successfully implemented, parent centric, audiologist-driven NHS program, it has certain limitations. The surveillance and the follow-up program for the babies that reported “Pass” results for the initial NHS was limited in its scope. Further, it focused on healthy newborn babies without including the high-risk infants. Selection bias does exist, as parents that did not opt for NHS were not included in the study.

### 4.3. Future Directions

Future studies may need to focus on both categories to track newborn babies who reported initial “Pass” and “Fail” results. It would be possible to include high-risk infants as well as babies who require extensive stay in NICU. These babies need to be assessed and monitored for their hearing development through systematic processes such as ABR and OAE.

## 5. Conclusion

The implementation of an NHS program is essential for early identification of hearing loss in newborn babies. Current study was an attempt to report the emerging data from the implementation of an NHS program. The study findings emphasize the need for a two-stage model of NHS. Further, the current study highlights the importance of parental engagement to increase the awareness regarding timely NHS, follow-up NHS, and hearing loss in children in the UAE.

## Figures and Tables

**Table 1:** Initial and follow-up NHS results.

	NHS-pass (*n* = 1821)		NHS-fail (*n* = 1821)	
	Right *n* (%)	Left *n* (%)	Right *n* (%)	Left *n* (%)
NHS-initial	1592 (87.40%)	1627 (89.30%)	229 (12.60%)	194 (10.70%)
NHS-follow-up	399 (94.30%)	399 (94.30%)	24 (05.70%)	24 (05.70%)

^∗^NHS: newborn hearing screening.

**Table 2:** Patterns of diagnosed hearing loss.

	Bilateral		Unilateral		Total
	Females	Males	Females	Males	
SNHL	03	06	-	-	09
Maturation delay	05	03	03	01	12
ANSD	02	01	-	-	03
Total	10	10	03	01	24

^∗^SNHL: sensorineural hearing loss; ANSD: auditory neuropathy spectrum disorder.

**Table 3:** McNemar's chi-square test results for the right ear.

		Follow-up			
		Pass	Fail	Total	*p* value
Baseline	Pass	194	0	194	<0.001
	Fail	205	24	229	

	Total	399	24	423	

**Table 4:** McNemar's chi-square test results for the left ear.

		Follow-up			
		Pass	Fail	Total	*p* value
Baseline	Pass	229	0	229	<0.001
	Fail	170	24	194	

	Total	399	24	423	

## Data Availability

Data is available upon request. Please contact mohdayas@gmail.com if more information is required.
